# Multidrug-Resistant and Methicillin-Resistant *Staphylococcus aureus* (MRSA) in Hog Slaughter and Processing Plant Workers and Their Community in North Carolina (USA)

**DOI:** 10.1289/ehp.1306741

**Published:** 2014-02-07

**Authors:** Ricardo Castillo Neyra, Jose Augusto Frisancho, Jessica L. Rinsky, Carol Resnick, Karen Colleen Carroll, Ana Maria Rule, Tracy Ross, Yaqi You, Lance B. Price, Ellen Kovner Silbergeld

**Affiliations:** 1 Department of Epidemiology, and; 2 Department of Environmental Health Sciences, Johns Hopkins Bloomberg School of Public Health, Baltimore, Maryland, USA; 3 Department of Epidemiology, University of North Carolina at Chapel Hill, Chapel Hill, North Carolina, USA; 4 Department of Pathology, Division of Microbiology, Johns Hopkins Hospital, Maryland, USA; 5 Department of Environmental and Occupational Health, George Washington University, Washington, DC, USA

## Abstract

Background: Use of antimicrobials in industrial food-animal production is associated with the presence of antimicrobial-resistant *Staphylococcus aureus* (*S. aureus*) among animals and humans. Hog slaughter/processing plants process large numbers of animals from industrial animal operations and are environments conducive to the exchange of bacteria between animals and workers.

Objectives: We compared the prevalence of multidrug-resistant *S. aureus* (MDRSA) and methicillin-resistant *S. aureus* (MRSA) carriage among processing plant workers, their household members, and community residents.

Methods: We conducted a cross-sectional study of hog slaughter/processing plant workers, their household members, and community residents in North Carolina. Participants responded to a questionnaire and provided a nasal swab. Swabs were tested for *S. aureus*, and isolates were tested for antimicrobial susceptibility and subjected to multilocus sequence typing.

Results: The prevalence of *S. aureus* was 21.6%, 30.2%, and 22.5% among 162 workers, 63 household members, and 111 community residents, respectively. The overall prevalence of MDRSA and MRSA tested by disk diffusion was 6.9% and 4.8%, respectively. The adjusted prevalence of MDRSA among workers was 1.96 times (95% CI: 0.71, 5.45) the prevalence in community residents. The adjusted average number of antimicrobial classes to which *S. aureus* isolates from workers were resistant was 2.54 times (95% CI: 1.16, 5.56) the number among isolates from community residents. We identified two MDRSA isolates and one MRSA isolate from workers as sequence type 398, a type associated with exposure to livestock.

Conclusions: Although the prevalence of *S. aureus* and MRSA was similar in hog slaughter/processing plant workers and their household and community members, *S. aureus* isolates from workers were resistant to a greater number of antimicrobial classes. These findings may be related to the nontherapeutic use of antimicrobials in food-animal production.

Citation: Castillo Neyra R, Frisancho JA, Rinsky JL, Resnick C, Carroll KC, Rule AM, Ross T, You Y, Price LB, Silbergeld EK. 2014. Multidrug-resistant and methicillin-resistant *Staphylococcus aureus* (MRSA) in hog slaughter and processing plant workers and their community in North Carolina (USA). Environ Health Perspect 122:471–477; http://dx.doi.org/10.1289/ehp.1306741

## Introduction

*Staphylococcus aureus* (*S. aureus*) is an important pathogen that can cause serious and life-threatening infections in humans. Clinical problems caused by *S. aureus* range from localized illnesses, such as necrotizing skin infections and folliculitis, to systemic diseases, including toxic shock syndrome (Lowy 1998). *S. aureus* infections have become more dangerous and costly to treat over the past 20 years because of increasing prevalence of antimicrobial resistance. Of considerable concern is methicillin-resistant *S. aureus* (MRSA), as well as multidrug-resistant *S. aureus* (MDRSA) (Gordon and Lowy 2008). Several studies in hospitals in the United States have reported that MRSA is the most common cause of skin and soft tissue infections (King et al. 2006; Moran et al. 2006; Parchman and Munoz 2009), and MRSA carriage is associated with subsequent infection and increased morbidity and mortality compared with noncarriage (Datta and Huang 2008).

*S. aureus* colonizes skin and can persist in the nares; positive nasal carriage is indicative of exposure and is associated with an increased risk of clinical infection in hospitalized populations (Davis et al. 2004; Stevens et al. 2010). Based on risk factors associated with exposure, MRSA strains are often classified as health care–associated MRSA (HA-MRSA), or community-associated MRSA (CA-MRSA). Since 2001, the increases in MRSA exposures and infections in the United States are largely due to community-associated strains, such that MRSA can no longer be controlled solely on the basis of measures implemented within health care settings (Como-Sabetti et al. 2009; Stefani et al. 2012).

Within the category of CA-MRSA, studies in several countries have identified specific strains associated with livestock and which have been termed livestock-associated MRSA (LA-MRSA) (Armand-Lefevre et al. 2005; Bisdorff et al. 2012; DeBoer et al. 2009; Ogata et al. 2012; Smith and Pearson 2011; Waters et al. 2011). Studies have reported increased risks of MRSA carriage among persons working with livestock, including swine (Aubry-Damon et al. 2004; Denis et al. 2009; Geenen et al. 2012; Morcillo et al. 2012; Mulders et al. 2010; Nijsten et al. 1996; Voss et al. 2005); among veterinarians treating livestock (Garcia-Graells et al. 2012; Hanselman et al. 2006); and, more recently, among persons without direct livestock contact but residing in areas of high livestock density (Feingold et al. 2012). In addition, several recent studies have reported on the prevalence of MDRSA carriage among livestock, farm workers, and slaughterhouse workers (Khanna et al. 2008; Oppliger et al. 2012; Smith and Pearson 2011; VanCleef et al. 2010).

In comparison with the European Union, relatively fewer studies examining MDRSA and MRSA exposures in hog production have been conducted in the United States (Leedom Larson et al. 2010; Osadebe et al. 2013; Rinsky et al. 2013; Smith et al. 2009) and, to our knowledge, no studies have been published examining the prevalence of MRSA among workers in U.S. hog slaughter and processing plants or the household members of these workers. Therefore, we undertook a study of workers in a large hog slaughter and processing plant, their household members, and community residents. The objective of our study was to test the hypothesis that workers have a higher prevalence of carriage of nonsusceptible strains of *S. aureus*, including MDRSA and MRSA, compared with residents in the same area who do not work in hog slaughter and processing. We also tested the hypothesis that workers are more likely to carry *S. aureus* isolates that are resistant to more antimicrobials as compared with community residents from the same area. We included household members in this study based on studies of household transmission of *S. aureus* and MRSA that reported transmission rates within households as high as 43% (Davis et al. 2012). We hypothesized that household members of workers would also have greater exposure to nonsusceptible strains of *S. aureus* than community referents.

## Methods

*Study design and recruitment*. We conducted a cross-sectional study between September and November 2011 in Tar Heel, North Carolina, the location of the Smithfield plant, the largest hog slaughter and processing plant in the United States. Tar Heel is sparsely populated [117 residents, according to the 2010 U.S. Census (U.S. Census Bureau 2011)], with most workers and community referents residing in nearby cities and towns in southern North Carolina and northern South Carolina. The workforce at the Tar Heel plant included approximately 4,500 workers and was unionized, which facilitated enrollment of workers in the study. Study participants were recruited through outreach efforts by our partner, the United Food and Commercial Workers International Union (UFCW) local 1208. Prior to data collection, we met with local and national officials of the UFCW, as well as with shop stewards of the local union (employees who represent the union at each work area within the plant). These individuals informed the union membership about the study. We asked workers to invite up to two members of their community (people who lived nearby, but who did not live with them or work at the plant), and up to two people living with them who did not work at the plant. Through these efforts we enrolled three categories of participants: *a*) plant workers, *b*) household members of plant workers (up to two per worker), and *c*) community residents. All data collection activities were conducted at the union office, located within one mile of the plant. Before initiating the study, we informed Smithfield about the study through telephone contact with the Vice President for Environmental Affairs.

Participant enrollment took place between Thursdays and Sundays in three waves. All workers had been at work within the past week and many came directly from work. Prior to enrollment, a verbal screening was conducted to determine eligibility of persons approaching the enrollment sessions: all participants were required to be ≥ 18 years of age, able to speak and understand either English or Spanish, reside in the local area (for community residents) defined as southern North Carolina and northern South Carolina, and were not working at a health care facility. Those who met these inclusion criteria were assigned a unique participant code and were directed to interview stations where oral informed consent was obtained prior to data collection. No personal identifiers were collected in order to protect confidentiality. The study was reviewed and approved by the Johns Hopkins Bloomberg School of Public Health Committee on Human Research.

*Data collection and biological sampling*. An extensive interview was conducted using a standardized questionnaire to collect information on demographic data, current and past occupational history, recent health history (including infections and any use of antimicrobials), contact with live animals (livestock and companion animals), and typical diet. Fluent English/Spanish speakers administered the questionnaire in both languages. We pretested the questionnaire in English and Spanish for clarity and consistency on six non-Hispanic and six Hispanic union members.

After completing the questionnaire, trained personnel collected a swab sample [BD Diagnostic Systems, (Sparks, MD) dual swab with Amies agar gel] from both nares of each participant. The rayon-tipped swab applicator was then placed into its plastic tube containing transport medium. The transport tube was labeled with the participant code, and shipped to our laboratory at Johns Hopkins by express courier service.

*Microbiological and molecular analyses*. Upon arrival at the laboratory, all samples were kept at room temperature until they were processed by the Johns Hopkins Hospital Laboratory of Medical Microbiology, within 72 hr of collection. Nasal swabs were cultured on BBL 5% sheep blood agar (SBA) and CHROMAgar Staph aureus plates (both from BD Diagnostic Systems) and incubated aerobically at 35°C for ≤ 48 hr before reading. Any suspected colony (β-hemolytic on 5% SBA or mauve colored on ChromAgar Staph aureus plates) was further subjected to Gram staining and the catalase assay and slide agglutination test (Rabbit Coagulase Plasma; ProLab, Richmond Hill, Ontario, Canada). Gram-positive cocci in clusters that were catalase positive and coagulase positive were identified as *S. aureus* (Becker and von Eiff 2011) and subcultured on 5% SBA to isolate pure colonies before being transferred into 30% glycerol and frozen at –80°C.

We transferred one isolate from each *S. aureus*-positive culture to our laboratory for antimicrobial susceptibility testing using the disk diffusion method [Clinical and Laboratory Standards Institute (CLSI) 2008]. Isolates were first regrown in Mueller Hinton broth and then examined for susceptibility to cefoxitin, ciprofloxacin, clindamycin, erythromycin, gentamicin, sulfamethoxazole/trimethoprim, quinupristin/dalfopristin, and tetracycline. We used the zone of growth inhibition around specific-antibiotic disks to assess the minimum inhibitory concentration (MIC). Based on these MICs and according to CLSI (2008) standards, we classified the isolates as susceptible, intermediate, or resistant to each antimicrobial except for cefoxitin, for which isolates were classified as either susceptible or resistant. Cefoxitin-resistant isolates were identified as phenotypic MRSA because resistance to cefoxitin predicts resistance to methicillin (Fernandes et al. 2005; Magiorakos et al. 2012).

We performed polymerase chain reaction (PCR) assays targeting *S. aureus nuc* (endonuclease) and *mecA* (penicillin-binding protein) genes, using the primers *nuc-1*: 5´-TCAG​CAAA​TGCA​TCAC​AAAC​AG-3´; *nuc-2*: 5´-CGTA​AATG​CACT​TGCT​TCAG​G-3´; *mecA*-1: 5´-GGGA​TCAT​AGCG​TCAT​TATT​C-3´ and *mecA*-2: 5´-AACG​ATTG​TGAC​ACGA​TAG​CC-3´ and methods previously reported (Poulsen et al. 2003). We defined as genotypic MRSA those specimens that were positive for the *mecA* gene. Because of variation in *mecA* sequences (Fluit 2011; García-Álvarez et al. 2011; Hanssen et al. 2004) that could lead to false negatives, we examined both phenotypically and genotypically characterized MRSA in our analyses. We performed multilocus sequence typing (MLST) of the seven housekeeping genes to identify *S. aureus* genetic strains as described by Enright et al. (2000).

*Statistical analysis*. The distributions of demographic, exposure, and outcome variables were examined and compared across the three categories of participants (workers, household members, community residents). As noted above, we classified isolates as either susceptible or resistant to cefoxitin; and as susceptible, intermediate, or resistant to other antimicrobials on the basis of MIC values (CLSI 2008). In addition, we also classified the isolates as either susceptible or nonsusceptible (the latter category including both intermediate and resistant isolates) as proposed by Magiorakos et al. (2012). Consistent with Magiorakos et al. (2012), we classified isolates as MDRSA if they were nonsusceptible to ≥ 3 classes of antimicrobials or were MRSA (i.e., resistant to cefoxitin). Although the susceptible and nonsusceptible categories may be more important for epidemiological purposes (Magiorakos et al. 2012) the CLSI definition is reliable in determining therapeutic failure (Kahlmeter et al. 2003). To facilitate comparison to the clinical literature, we examined both classifications.

The prevalence of *S. aureus*, nonsusceptible *S. aureus*, MDRSA, and MRSA was determined for each participant group and for the study population as a whole. We also determined the proportions of *S. aureus* isolates that were nonsusceptible, MDRSA, and MRSA among participants with positive *S. aureus* swabs. Depending on the number of individuals in each category, we used chi-squared or Fisher’s exact tests to compare proportions across participant categories.

We used unadjusted and adjusted Poisson regression to compare the average number of antimicrobials to which *S. aureus* isolates were resistant (based on the CLSI definition) among workers, household members, and community residents. We also used unadjusted and adjusted log binomial regression models to compare the prevalence of MDRSA among workers, household members, and community residents. All multivariable models were adjusted for age (in groups), any self-reported use of antimicrobials in the previous 6 months (yes/no), and any self-reported visit to a medical facility in the previous 6 months (yes/no). A medical facility was defined as any place where medical care is provided, including hospitals, clinics, doctor offices, and nursing homes. The variables included in the adjusted models were selected based on *a priori* assumptions.

Finally, we examined the patterns of antimicrobial resistance found in the *S. aureus* isolates and the distribution of *S. aureus* and genotypic MRSA strains based on MLST analysis. All statistical analyses were performed using Stata version 11 (StataCorp, College Station, TX), with a significance level of 0.05.

## Results

*Study population*. We enrolled 336 participants. Of those, 162 participants were hog slaughter/processing plant workers, 63 were household members from 50 different households, and 111 were community residents.

Community residents were more often white non-Hispanic (18%) than workers (3.1%) or their household members (1.6%) (*p* < 0.01) (Table 1). On average, workers were older than household members or community residents [mean = 41 vs. 38.6 and 32.3 years of age, respectively; analysis of variance, F(2,2) = 9.01, *p* < 0.01]. There were more women (58.5% overall) than men in each group, but there were no statistically significant differences among groups with regard to sex, visit to a medical facility or using antimicrobials in the last 6 months, having a MRSA diagnosis in the past year, or animal contact at home unrelated to hog slaughter and processing work.

**Table 1:** Study population characteristics by participant category.

Category	Total [*n *= 336 (%)]	Worker [*n *= 162 (%)]	Household member [*n *= 63 (%)]	Community resident [*n *= 111 (%)]	χ^2^ test statistic (df)	*p*-Value
Age (years)					48.13 (10)	< 0.01
18–25	89 (26.5)	24 (14.8)	31 (49.2)	34 (30.6)
26–35	66 (19.6)	32 (19.8)	10 (15.9)	24 (21.6)
36–45	65 (19.3)	40 (24.7)	7 (11.1)	18 (16.2)
46–55	62 (18.5)	43 (26.5)	6 (9.5)	13 (11.7)
56–82	50 (14.8)	23 (14.2)	8 (12.7)	19 (17.1)
Sex, female	196 (58.5)	88 (54.7)	41 (65.1)	67 (60.4)	2.26 (1)	0.32
Race/ethnicity					31.07 (6)	< 0.01
African American	231 (68.8)	114 (70.4)	46 (73.0)	71 (64.0)
Hispanic	52 (15.5)	30 (18.5)	13 (20.6)	9 (8.1)
White non-Hispanic	26 (7.7)	5 (3.1)	1 (1.6)	20 (18.0)
Native American	18 (5.4)	9 (5.6)	2 (3.2)	7 (6.3)
Other	9 (2.7)	4 (2.5)	1 (1.6)	4 (3.6)
Animal contact on home property	161 (47.9)	74 (45.7)	28 (44.4)	59 (53.2)	1.85 (2)	0.42
Medical facility visit in last 6 months	193 (58.0)	89 (54.9)	40 (64.5)	64 (58.7)	1.73 (2)	0.42
MRSA diagnosis in the last year	3 (0.9)	2 (1.2)	1 (1.6)	0 (0.0)	—^*a*^	0.43
Use of anti­microbials in last 6 months	80 (23.8)	37 (22.8)	17 (27.0)	26 (23.4)	0.44 (2)	0.82
Prevalence
*S. aureus*	79 (23.5)	35 (21.6)	19 (30.2)	25 (22.5)	1.94 (2)	0.38
Non­susceptible *S. aureus*	65 (19.4)	28 (17.3)	13 (21.0)	24 (21.6)	0.88 (2)	0.65
MRSA phenotype^*b*^	16 (4.8)	9 (5.6)	3 (4.8)	4 (3.6)	0.55 (2)	0.76
MRSA *mecA*^*c*^	9 (2.7)	5 (3.1)	2 (3.2)	2 (1.8)	—^*a*^	0.74
MDRSA^*d*^	23 (6.9)	13 (8.0)	4 (6.5)	6 (5.4)	0.73 (2)	0.70
^***a***^*p*-Value was calculated with Fisher’s exact test. ^***b***^Phenotypic MRSA defined as *S. aureus* resistant to cefoxitin. ^***c***^MRSA identified by detection of the *mecA* gene, genotypic MRSA is a subset of that detected phenotypically. ^***d***^MDRSA denotes *S. aureus* non­susceptible to three or more of the anti­microbials used in this study or resistant to cefoxitin.						

*Prevalence of* S. aureus, *nonsusceptible* S. aureus, *MDRSA, and MRSA*. The overall prevalence of *S. aureus* nasal carriage among the study population was 23.5% (79/336) and was higher among household members (30.2%) than workers (21.6%) or community members (22.5%) (*p* = 0.38) (Table 1). We tested 78 isolates from the 79 *S. aureus*-positive participants for antimicrobial susceptibility (one isolate did not grow). The overall prevalence of nonsusceptible *S. aureus* was 19.4%, with similar prevalence between groups. The overall prevalence of MDRSA was 6.9% (23/335), with 8.0%, 6.5%, and 5.4% among workers, household members and community residents, respectively. The overall prevalence of phenotypic MRSA was 4.8% (16/335), with 5.6%, 4.8%, and 3.6% among workers, household members and community residents, respectively. Nine of the 16 phenotypic MRSA isolates were positive for *mecA*, providing an overall prevalence of genotypic MRSA of 2.7% (9/335); with a prevalence of 3.1%, 3.2%, and 1.8% among workers, household members and community residents, respectively.

*Proportion of nonsusceptible* S. aureus, *MDRSA, and MRSA in* S. aureus *isolates*. The proportion of *S. aureus* isolates (*n* = 78) that were nonsusceptible to at least one antimicrobial was higher in community members (96.0%) than workers (80.0%) or household members (72.2%) (*p* = 0.09) (Table 2). The proportion of MDRSA among all *S. aureus* isolates was higher in isolates from workers (37.1%) than household members (22.2%) or community residents (24.0%) (*p* = 0.41), and the proportion of phenotypic MRSA also was higher in workers (25.7%) than household members (16.7%) or community residents (16.0%) (*p* = 0.67). The proportion of *mecA*-positive MRSA was 14.3%, 11.1%, and 8% among workers, household members and community residents, respectively. The prevalence of MDRSA and MRSA in *S. aureus* isolates was similar between household members and community residents.

**Table 2:** Distribution of non­susceptibility, multidrug-resistance, and MRSA among those positive for *S. aureus*.

Classification	Total [*n *= 78 (%)]	Worker [*n *= 35 (%)]	Household member [*n *= 18 (%)]	Community resident [*n *= 25 (%)]	*p*-Value^*a*^
Non­susceptible *S. aureus*^*b*^	65 (83.3)	28 (80.0)	13 (72.2)	24 (96.0)	0.09
MRSA phenotype^*c*^	16 (20.5)	9 (25.7)	3 (16.7)	4 (16.0)	0.67
MRSA *mecA*^*d*^	9 (11.5)	5 (14.3)	2 (11.1)	2 (8.0)	0.90
MDRSA^*e*^	23 (29.5)	13 (37.1)	4 (22.2)	6 (24.0)	0.41
^***a***^*p*-Value calculated with Fisher’s exact test. ^***b***^*S. aureus* intermediate or resistant to any anti­microbial class. ^***c***^Phenotypic MRSA defined as *S. aureus* resistant to cefoxitin. ^***d***^MRSA identified by detection of *mecA* gene, genotypic MRSA is a subset of that detected phenotypically. ^***e***^MDRSA denotes *S. aureus* non­susceptible to three or more of the anti­microbials used in this study or resistant to cefoxitin.					

**Figure 1:**
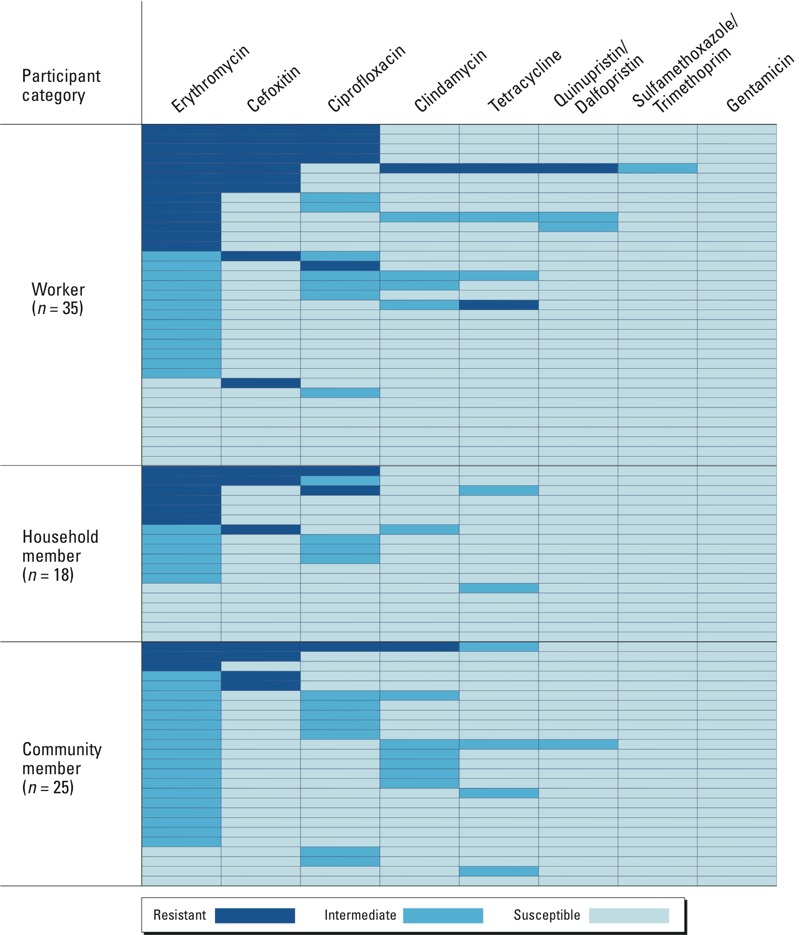
Heat map showing the pattern of anti­microbial resistance of the 78 isolates of *S. aureus*. Each row represents one isolate tested for susceptibility from a *S. aureus*–positive participant. Antimicrobial resistance was assessed by disk diffusion and cutoffs defined by CLSI (2008) guidelines; resistance to cefoxitin was classified as either susceptible or resistant, based on CLSI (2008) guidelines.

*Antimicrobial resistance profile of* S. aureus. We also examined the distribution of susceptible, intermediate, and resistant isolates and found unequal proportions across participant groups (Fisher’s exact test, *p* < 0.01). Proportions extracted from Figure 1 show that among participants carrying *S. aureus*, workers had the highest proportion of *S. aureus* resistant to at least one antimicrobial class (48.6%; 17/35), followed by household members (38.9%; 7/18) and community residents (20.0%; 5/25). The highest proportion of *S. aureus* showing intermediate resistance to at least one antimicrobial class was found in community members (76.0%; 19/25), followed by household members (33.3%; 6/18) and workers (31.4%; 11/35).

Detailed resistance profiles of these isolates (Figure 1) suggest that the numbers of different classes of antimicrobials to which *S. aureus* isolates were resistant varied among the participant groups. Workers carried *S. aureus* that were resistant to more antimicrobials compared with isolates carried by household members or community residents. Isolates from community residents were more likely to have intermediate resistance than isolates from workers or household members. The patterns of resistance to specific antimicrobials also varied among groups. Erythromycin nonsusceptibility (resistant or intermediate) was the most common phenotype observed in all groups. Workers and household members had the highest prevalence of erythromycin-resistant *S. aureus* (Figure 1). The most common pattern of multiple resistance in the entire study population was nonsusceptibility to erythromycin and ciprofloxacin (14.1%; 11/78), followed by nonsusceptibility to erythromycin, cefoxitin, and ciprofloxacin (9%; 7/78) and nonsusceptibility to erythromycin and cefoxitin (6.4%; 5/78).

*Group differences in* S. aureus *antimicrobial resistance*. Compared with isolates from community residents, isolates from workers and household members were on average resistant to 2.54 times (95% CI: 1.16, 5.56) and 1.69 times (95% CI: 0.64, 4.46) more antimicrobial classes, respectively, after adjusting for age, visiting a medical facility in the last 6 months, or using antimicrobials in the last 6 months (Table 3). Age, visiting a medical facility in the last 6 months, and taking antimicrobials in the last 6 months were not significantly associated with the number of antimicrobial classes to which the isolates were resistant and did not confound the associations with working in a hog-processing facility.

**Table 3:** Unadjusted and adjusted estimates of the association between exposures and the mean number of anti­microbials classes to which a *S. aureus* isolate was resistant.

Category	*n*	Unadjusted mean ratio (95% CI)	*p*-Value	Adjusted mean ratio (95% CI)	*p*-Value
Participant group
Community resident	25	Referent	—	Referent	—
Household member	18	1.70 (0.70, 4.10)	0.24	1.69 (0.64, 4.46)	0.29
Worker	35	2.46 (1.17, 5.17)	0.17	2.54 (1.16, 5.56)	0.02
Age (years)
18–25	29	Referent	—	Referent	—
26–35	17	1.93 (0.97, 3.87)	0.06	1.67 (0.80, 3.46)	0.17
36–45	12	1.13 (0.46, 2.77)	0.79	1.10 (0.43, 2.78)	0.85
46–55	11	1.05 (0.41, 2.72)	0.91	0.78 (0.28, 2.20)	0.64
56–82	8	1.45 (0.56, 3.74)	0.44	1.14 (0.43, 3.08)	0.79
Medical facility visit in last 6 months^*a*^	39	1.33 (0.75, 2.36)	0.33	1.37 (0.75, 2.48)	0.31
Use of antimicrobials in last 6 months^*b*^	19	0.85 (0.44, 1.66)	0.64	0.93 (0.47, 1.85)	0.83
^***a***^Reference group are those who did not visit a medical facility in last 6 months. ^***b***^Reference group are those who did not take anti­microbials in last 6 months.					

The prevalence of MDRSA carriage in workers was 1.96 times higher (95% CI: 0.71, 5.45) than in community residents after adjusting for other variables (*p* = 0.20) (Table 4). The prevalence of MDRSA in household members was comparable to community residents [prevalence ratio (*PR*) = 1.04; 95% CI: 0.25, 4.28].

**Table 4:** Unadjusted and adjusted PRs estimating the association between exposures and carriage of multidrug-resistant *S. aureus*.

Category	*n*	Unadjusted PR (95% CI)	*p*-Value	Adjusted PR (95% CI)	*p*-Value
Participant group
Community resident	111	Referent	—	Referent	—
Household member	62	1.19 (0.35, 4.07)	0.78	1.04 (0.25, 4.28)	0.96
Worker	162	1.48 (0.58, 3.79)	0.41	1.96 (0.71, 5.45)	0.20
Age (years)
18–25	88	Referent		Referent	—
26–35	66	1.33 (0.45, 3.95)	0.60	0.97 (0.30, 3.15)	0.96
36–45	65	0.68 (0.18, 2.61)	0.57	0.54 (0.14, 2.17)	0.39
46–55	62	0.95 (0.28, 3.21)	0.93	0.55 (0.14, 2.22)	0.40
56–82	50	1.17 (0.35, 3.96)	0.80	1.07 (0.31, 3.74)	0.91
Medical facility visit in last 6 months^*a*^	193	0.96 (0.42, 2.22)	0.92	0.98 (0.41, 2.32)	0.96
Use of antimicrobials in last 6 months^*b*^	80	0.89 (0.34, 2.31)	0.80	1.07 (0.40, 2.86)	0.90
PR, prevalence ratio. ^***a***^Reference group are those who did not visit a medical facility in last 6 months. ^***b***^Reference group are those who did not take anti­microbials in last 6 months.					

**Figure 2:**
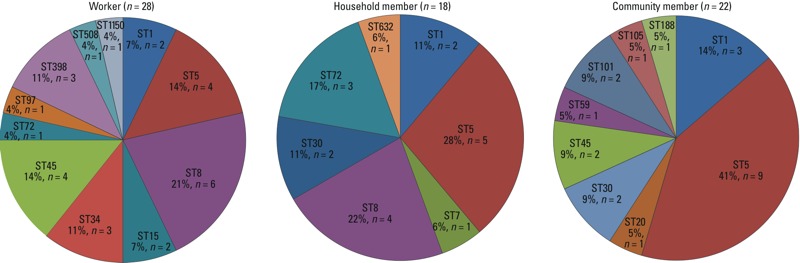
*S. aureus* sequence type diversity and distribution. Sequence types were based on seven housekeeping genes that were derived from whole genome sequences of each isolates.

*MLST and* S. aureus *strains by group*. We identified 19 unique sequence types (ST) from 68 *S. aureus* isolates (Figure 2). Sequence types for the 11 remaining isolates could not be determined. *S. aureus* isolates from workers demonstrated greatest sequence type diversity. ST1 and ST5 were found in all three participant groups. ST8 was common among *S. aureus* isolates from workers and household members (21% and 22%, respectively) but absent among isolates from community residents. ST72 was also observed only among isolates from workers (*n* = 1) and household members (*n* = 3). Notably, three isolates, all from workers, were identified as ST398, including two MDRSA isolates and one MRSA isolate. Among MRSA isolates, ST8 was the predominant sequence type (38%), followed by ST1 (19%).

## Discussion

To our knowledge, this is the first published study in the United States to examine carriage of *S. aureus*, MDRSA, and MRSA in hog slaughter and processing plant workers and their communities. Although the prevalence of *S. aureus* and MRSA was similar among all three participant groups, *S. aureus* isolates from workers were resistant to a greater number of antimicrobial classes than isolates carried by household members or community residents. Workers also had a higher prevalence of MDRSA than community residents, although the difference was not statistically significant. The overall prevalence of *S. aureus* in our population was 23.5%, which is slightly lower than the estimated prevalence in U.S. adults (27.4% for people 20–59 years of age) based on NHANES data for 2003–2004 (Gorwitz et al. 2008). However, the prevalence of MRSA in our population [4.8% based on CLSI (2008) criteria, 2.7% *mecA* positive] was higher than the NHANES estimate of 1.1%. The prevalence of MRSA carriage in our study was also greater than estimates from two studies of young, healthy, adult military recruits that reported prevalences of MRSA carriage between 0.5% and 2% (Findlay et al. 2010; Zinderman et al. 2004).

PCR using previously reported primers (Poulsen et al. 2003) did not detect *mecA* in 7/16 phenotypically characterized MRSA isolates, consistent with the presence of variant *mecA* genes that are not detected by standard probes (García-Álvarez et al. 2011; Petersen et al. 2013). Therefore, we reported both phenotypic and genotypic MRSA as suggested by Fluit (2011). We did not conduct further PCR analyses to identify any *mecA* variants. We looked for ST398, a strain variant of the clonal complex (CC) 398 that has been associated with exposure to hogs and other livestock (Armand-Lefevre et al. 2005; Feingold et al. 2012; Smith and Pearson 2011). Three ST398 isolates were identified in workers using MLST, including one that was MRSA, and two that were susceptible to methicillin (cefoxitin) but classified as MDRSA on the basis of resistance to ≥ 3 other antimicrobial classes. Studies in European countries have showed that pigs are a source of MRSA CC398 infections in humans, with the predominant ST being ST398 (Lewis et al. 2008), and that MRSA CC398 is much more prevalent among persons exposed to hogs than their family members or nonexposed community residents (Cuny et al. 2009; Oppliger et al. 2012; VanCleef et al. 2010). Similar to our results, a Swiss study of antimicrobial-resistant *S. aureus* in pigs and pig farmers reported that 22% of all MRSA and methicillin-susceptible *S. aureus* CC398 strains were multidrug resistant (Oppliger et al. 2012).

We observed evidence of greater *S. aureus* genotype diversity in isolates from workers (11 MLST sequence types) than in isolates from household members or community residents (7 and 9 sequence types respectively). Oppliger et al. (2012) reported more *S. aureus* genotype diversity in isolates from non-farmers than pig farmers. We identified ST5 in all three participant groups, ST8 in workers and household members, and ST398 in workers only. Similarly, a French study observed *S. aureus* ST5 in both pig farmers and non-farmers, and ST8 and ST398 in pig farmers only (Armand-Lefevre et al. 2005). ST1 was identified in isolates from all three groups in our study, and was the most common isolate identified in pork meat in a U.S. study (Waters et al. 2011). However, ST1 was not prevalent in pigs, pig farmers, or non-farmers in the Swiss study (Oppliger et al. 2012).

The most common *S. aureus* genotypes in hog slaughter and processing plant workers in our study were ST8 (belonging to CC8) and ST5 (belonging to CC5), with the predominant MRSA genotype being ST8 (4/9 isolates). In contrast, studies from other countries reported CC9 and CC398 as the predominant *S. aureus* and MRSA genotypes in pigs and pig farmers (Armand-Lefevre et al. 2005; Oppliger et al. 2012). ST8 and ST5 have been consistently reported to be the most common MRSA strains in isolates from pigs and pork in the United States (Molla et al. 2012; Pu et al. 2009; Waters et al. 2011). We did not identify ST9 (belonging to CC9) among *S. aureus* isolates, although this sequence type was previously found in pigs and pork in the United States (Molla et al. 2012; Waters et al. 2011).

Importantly, we found that, among participants carrying *S. aureus*, workers had the highest proportion of *S. aureus* resistant to at least one antimicrobial class. Moreover, workers had isolates resistant to more antimicrobial classes and also had a higher prevalence of carriage of MDRSA as compared with community residents. Multidrug resistance also was more pronounced in isolates from Swiss hog farmers than isolates from non-farmers (Oppliger et al. 2012).

Infections caused by multidrug resistant bacteria are associated with worse health outcomes and higher expenditures (Cardoso et al. 2012; Stone 2009); however, few studies have examined the prevalence of MDRSA in human populations in the United States. One previous North Carolina study reported a 16% prevalence of MDRSA carriage among industrial livestock operation workers compared with 9% among antibiotic-free livestock operation workers (Rinsky et al. 2013). The greater number of drugs to which isolates from workers in our study were resistant is also noteworthy and may be associated with the use of multiple antimicrobials in hog feeds (Silbergeld et al. 2008).

We found resistance to erythromycin was more prevalent than resistance to any other antimicrobial class, similar to Oppliger et al. (2012). However, patterns of resistance to other antimicrobials differed between the two studies, possibly reflecting differences in the use of antimicrobials as swine feed additives between the United States and Switzerland.

In the present study, we observed the prevalence of carriage of resistant strains of *S. aureus* to be greater in all studied groups than in the general U.S. population, but we did not observe differences between groups for some carriage outcomes. Although differences may have been obscured in part because of small sample sizes within groups, it is also possible that the non-worker groups in our study were exposed through environmental pathways from both farms and slaughter and processing operations. Studies by our group and others support this possibility. For example, *S. aureus* and MDRSA have been measured in air releases from intensive hog farms in the United States (Chapin et al. 2005; Gibbs et al. 2004, 2006), detected at distances of 150 m downwind from swine houses in Germany (Schulz et al. 2012), and found in hogs being transported in open trucks from farms to the slaughter house and in untreated swine house wastes and other releases (Burkholder et al. 2007). This explanation is also supported by other work by our group on clusters of MRSA infections among persons residing in areas of intensive hog production in the Netherlands and in northern North Carolina (Feingold et al. 2012).

The overall elevated rates of MDRSA and MRSA across participant groups, and the higher rate in the worker group, may be explained by the concentration of swine farms over the greater Tar Heel region and the common use of different antimicrobial formulations as growth promoters. The slaughterhouse plant in the present study served as a hub for collecting swine from these farms. As a result, workers at the Tar Heel plant were exposed to swine from different farms, and these animals may have carried strains of *S. aureu*s with different patterns of antimicrobial resistance. In contrast, non-workers, depending on where they lived, may have been indirectly exposed to relatively few farms and a less diverse set of *S. aureu*s strains.

## Conclusions

Our results raise concerns about the exposure of hog slaughter and processing plant workers to antimicrobial-resistant *S. aureus. S. aureus* isolates from workers were, on average, resistant to more classes of antimicrobials than isolates from community residents. In addition, among *S. aureus*–positive participants, a greater proportion of workers carried strains of *S. aureus* resistant to at least one antimicrobial class. Further, the overall prevalence of MRSA carriage identified in our study population in 2011 was higher than the estimate for the general U.S. population based on NHANES data for 2003–2004 (Gorwitz et al. 2008).

The observation of a similar higher prevalence of MRSA among all groups in our study may be in part related to nonoccupational exposures in the region, which has the highest density of industrial hog farms and hogs in the United States (Wing et al. 2000). Further studies will be crucial for the identification of factors associated with nonoccupational exposures.

Our results suggest a need for surveillance of antimicrobial-resistant *S. aureus* in populations with direct or indirect exposure to livestock. Finally, our study adds to concerns about the use of antimicrobials for nontherapeutic purposes as part of food-animal production, a practice thought to contribute to selection for antimicrobial-resistant strains of *S. aureus* in the community, especially in the food-production system.
